# Pembrolizumab plus either epacadostat or placebo for cisplatin-ineligible urothelial carcinoma: results from the ECHO-307/KEYNOTE-672 study

**DOI:** 10.1186/s12885-023-10727-3

**Published:** 2024-07-25

**Authors:** Andrea Necchi, Michiel S. Van der Heijden, Dmytro Trukhin, Avivit Peer, Howard Gurney, Boris Y. Alekseev, Francis X. Parnis, Raya Leibowitz, Maria De Santis, Petros Grivas, Jason Clark, Mihaela Munteanu, Ritesh Kataria, Calvin Jia, Arjun V. Balar, Ronald de Wit

**Affiliations:** 1 Vita-Salute San Raffaele University Milan, Milan, Italy; 2 Department of Medical Oncology, IRCCS San Raffaele Hospital and Scientific Institute, Via Olgettina 60, 20132 Milan, Italy; 3 The Netherlands Cancer Institute, Amsterdam, Netherlands; 4 Odessa Regional Oncological Dispensary, Odessa, Ukraine; 5 Rambam Health Care Center, Haifa, Israel; 6 Macquarie University, Sydney, Australia; 7 PA Hertsen Moscow Cancer Research Institute, Moscow, Russia; 8 Adelaide Cancer Centre, Kurralta Park, Australia; 9 University of Adelaide, Adelaide, Australia; 10 Oncology Institute, Shamir Medical Center, Be’er yaakov, Israel; 11 Sackler Faculty of Medicine, Tel Aviv University, Tel Aviv, Israel; 12 Department of Urology, Charité Universitätsmedizin, Berlin, Germany; 13 Medical University Vienna, Wien, Austria; 14 University of Washington, Fred Hutchinson Cancer Center, Seattle, Washington USA; 15 Incyte Corporation, Wilmington, Delaware USA; 16 Merck & Co., Inc., Rahway, NJ USA; 17 Laura and Isaac Perlmutter Cancer Center, NYU Langone Medical Center, New York, NY USA; 18 Erasmus MC Cancer Institute, Erasmus University Medical Center Rotterdam, Rotterdam, Netherlands

**Keywords:** IDO1, Epacadostat, PD-L1, PD1, Pembrolizumab, Urothelial carcinoma, Urinary tract neoplasms, Immune checkpoint inhibition, Immunotherapy, Randomized controlled study

## Abstract

**Background:**

Indoleamine 2,3- dioxygenase 1 (IDO1) is an immunosuppressive enzyme that has been correlated with shorter disease-specific survival in patients with urothelial carcinoma (UC). IDO1 may counteract the antitumor effects of immune checkpoint inhibitors. Epacadostat is a potent and highly selective inhibitor of IDO1. In the phase I/II ECHO-202/KEYNOTE-037 study, epacadostat plus pembrolizumab resulted in a preliminary objective response rate (ORR) of 35% in a cohort of patients with advanced UC.

**Methods:**

ECHO-307/KEYNOTE-672 was a double-blinded, randomized, phase III study. Eligible adults had confirmed locally advanced/unresectable or metastatic UC of the urinary tract and were ineligible to receive cisplatin-based chemotherapy. Participants were randomly assigned (1:1) to receive epacadostat (100 mg twice daily) plus pembrolizumab (200 mg every 3 weeks) or placebo plus pembrolizumab for up to 35 pembrolizumab infusions. The primary endpoint was investigator-assessed ORR per Response Evaluation Criteria in Solid Tumors (version 1.1).

**Results:**

A total of 93 patients were randomized (epacadostat plus pembrolizumab, *n* = 44; placebo plus pembrolizumab, *n* = 49). Enrollment was stopped early due to emerging data from the phase III ECHO-301/KEYNOTE-252 study. The median duration of follow-up was 64 days in both arms. Based on all available data at cutoff, ORR (unconfirmed) was 31.8% (95% CI, 22.46–55.24%) for epacadostat plus pembrolizumab and 24.5% (95% CI, 15.33–43.67%) for placebo plus pembrolizumab. Circulating kynurenine levels numerically increased from C1D1 to C2D1 in the placebo-plus-pembrolizumab arm and decreased in the epacadostat-plus-pembrolizumab arm. Epacadostat-plus-pembrolizumab combination treatment was well tolerated with a safety profile similar to the placebo arm. Treatment discontinuations due to treatment-related adverse events were more frequent with epacadostat (11.6% vs. 4.1%).

**Conclusions:**

Treatment with epacadostat plus pembrolizumab resulted in a similar ORR and safety profile as placebo plus pembrolizumab in cisplatin-ineligible patients with previously untreated locally advanced/unresectable or metastatic UC. At a dose of 100 mg twice daily, epacadostat did not appear to completely normalize circulating kynurenine levels when administered with pembrolizumab. Larger studies with longer follow-up and possibly testing higher doses of epacadostat, potentially in different therapy settings, may be warranted.

**Trial registration:**

ClinicalTrials.gov identifier: NCT03361865, retrospectively registered December 5, 2017.

**Supplementary Information:**

The online version contains supplementary material available at 10.1186/s12885-023-10727-3.

## Background

Cisplatin-based chemotherapy has been a standard first-line treatment for patients with locally advanced/unresectable or metastatic urothelial carcinoma (UC) for many years and remains an important component of care in the era of immunotherapy. For patients with locally advanced or metastatic UC without disease progression on first-line cisplatin- or carboplatin-based chemotherapy, recent data from the randomized, phase III JAVELIN Bladder 100 trial showed that maintenance treatment with the programmed death-ligand 1 (PD-L1) inhibitor avelumab plus best supportive care significantly improved overall survival (OS) compared with best supportive care alone [1]. Based on these results, this switch maintenance treatment has been approved in the United States (US), [2] and first-line cisplatin-based chemotherapy followed by avelumab maintenance is a new preferred regimen for cisplatin-eligible patients [3].

More than half of patients are cisplatin-ineligible due to poor performance status, renal dysfunction, and/or the presence of comorbidities [4, 5]; chemotherapy-related toxicity is also a concern. Carboplatin-based chemotherapy is a conventionally used alternative for cisplatin-ineligible patients [3, 6–8] and can be used with maintenance avelumab maintenance in the absence of disease progression, [1, 3] but these regimens are associated with lower response rates relative to cisplatin-based chemotherapy [9].

The immune checkpoint inhibitors atezolizumab or pembrolizumab can also be used in the first-line treatment of cisplatin-ineligible patients with UC whose tumors express PD-L1 based on the companion assay [3, 6, 7] or those who are not candidates for any platinum-based regimen (irrespective of PD-L1 status; certain countries such as the US only) [3]. However, only about one-quarter of cisplatin-ineligible patients with UC respond to single-agent atezolizumab or pembrolizumab [10, 11], although a higher objective response rate (ORR; 47%) has been observed in pembrolizumab-treated patients with PD-L1–positive tumors [12]. Thus, there remains a need for first-line treatment strategies that can increase the number of cisplatin-ineligible patients with advanced UC who benefit from immunotherapy.

Because cancer cells can exploit multiple mechanisms to evade the immune system [13], combination immunotherapy has the potential to enhance antitumor activity. Indoleamine 2,3- dioxygenase 1 (IDO1) is a tryptophan-catabolizing enzyme whose expression can be up-regulated by interferon [14]. IDO1 contributes to the immunosuppression of the tumor microenvironment [15], and elevated levels of IDO1 have been correlated to shorter survival in ovarian and endometrial cancers, as well as UC [16–18]. IDO1 has also been shown to blunt the activity of immune checkpoint inhibitors [19]. IDO1 and PD-L1 are co-expressed in a number of cancers [20–23], and preclinical studies have demonstrated the additive or synergistic effects of combined inhibition of IDO1 and PD-L1 [19, 24]. Therefore, we hypothesized that inhibiting IDO1 may augment the antitumor activity of immune checkpoint inhibitors in advanced UC.

Epacadostat, a potent and highly selective inhibitor of IDO1, has been shown to normalize levels of circulating kynurenine in patients with advanced solid malignancies when administered as monotherapy twice daily at doses of 100 mg or higher [25]. In the phase I/II ECHO-202/KEYNOTE-037 study (NCT02178722), epacadostat plus pembrolizumab resulted in a preliminary ORR of 35% (13/37) and was generally well tolerated in a cohort of patients with advanced UC [26]. Based on these encouraging results, the phase III ECHO-307/KEYNOTE-672 study, which compared epacadostat plus pembrolizumab with placebo plus pembrolizumab in cisplatin-ineligible patients with advanced UC, was undertaken to determine if efficacy could be improved with the combination.

## Methods

### Study design and participants

ECHO-307/KEYNOTE-672 (NCT03361865) was an international, placebo-controlled, double-blinded, randomized, phase III study. Eligible adults (≥ 18 years) had confirmed locally advanced/unresectable or metastatic UC of the urinary tract that was measurable per Response Evaluation Criteria in Solid Tumors (RECIST) version 1.1 [27], were ineligible to receive cisplatin-based therapy (eg, Eastern Cooperative Oncology Group performance status of 2 within 14 days before randomization, creatinine clearance between ≥ 30 and < 60 mL/min), had not received prior systemic chemotherapy for advanced UC (patients who received neoadjuvant or adjuvant platinum-containing chemotherapy and experienced recurrence > 12 months from completion of that chemotherapy were permitted), and provided tumor tissue for the central analysis of PD-L1. Exclusion criteria included disease suitable for local therapy with curative intent, known additional malignancy that is progressing or has required active treatment in the previous 3 years, active CNS metastases and/or carcinomatous meningitis, and active autoimmune disease requiring systemic treatment in the previous 2 years.

### Treatment and procedures

Participants were randomly assigned (1:1) to receive epacadostat plus pembrolizumab or placebo plus pembrolizumab for up to 35 pembrolizumab infusions (approximately 2 years) or until disease progression, unacceptable toxicity, or another study withdrawal criterion was met. Randomization was stratified by Bajorin risk score (0 vs. 1 vs. 2) and PD-L1 expression (combined positive score [CPS] per immunohistochemistry ≥ 10 vs. < 10). Intravenous pembrolizumab 200 mg was administered every 3 weeks, and epacadostat (or matching placebo) 100 mg was given orally twice daily. On day 1 of cycles 1 and 2 (C1D1 and C2D1, respectively), blood for serum pharmacodynamics analyses of kynurenine was drawn pre-dose from patients while they were in a fasting state.

### Study conduct

The study was initiated on December 4, 2017. On May 2, 2018, a strategic decision was made to permanently stop enrollment. The study was subsequently unblinded after the last patient completed the week 9 imaging assessment for efficacy analysis. The strategic decision to discontinue enrollment occurred after the phase III ECHO-301/KN-252 study did not show clinical benefit of combining epacadostat (100 mg twice daily) with pembrolizumab compared with placebo plus pembrolizumab in patients with advanced melanoma. The decision to stop enrollment was not based on new safety concerns observed in this study.

ECHO-307/KEYNOTE-672 was conducted in compliance with the Declaration of Helsinki, the International Council on Harmonization Guidelines for Good Clinical Practice, and applicable national and local regulatory requirements. The study protocol was approved by the Independent Ethics Committee/Institutional Review Board at each participating site, and all patients provided written informed consent.

### Endpoints

The original dual primary endpoints of ECHO-307/KEYNOTE-672 were progression-free survival (PFS) per independent central review and OS. A protocol amendment, initiated when enrollment was stopped, changed the primary endpoint to investigator-assessed ORR per RECIST version 1.1. ORR was defined as the proportion of patients with best response of complete response or partial response. Safety was assessed throughout the study, with adverse events (AEs) coded per Medical Dictionary for Regulatory Activities version 21.0 and graded per Common Terminology Criteria for Adverse Events version 4.03. The tertiary and exploratory objectives were estimation of efficacy by PD-L1 expression, evaluation of the pharmacodynamics of epacadostat as assessed by evaluating change from baseline in circulating kynurenine, evaluation of the pharmacokinetics of epacadostat, and identification of molecular biomarkers.

### Statistics

The original target enrollment was 650 patients, but when enrollment was stopped, the target was revised to 100 participants. The analysis population for efficacy analyses was the intention-to-treat population (ie, all randomized patients).

ORR was determined for each treatment group; the corresponding 95% confidence intervals were calculated using the Clopper-Pearson exact method. Although study efficacy procedures (including imaging) were discontinued after week 9 (first on-study imaging), a number of patients had completed scans beyond week 9 at the time that enrollment was terminated. Thus, ORR was assessed in two ways: based on investigator assessments using all available scans at the time of cutoff, as well as only those data collected at week 9.

The safety analysis population was the all participants as treated population (ie, all randomized patients who received ≥ 1 dose of study treatment). Safety outcomes were summarized using descriptive statistics.

The pharmacodynamics analysis population for evaluation of circulating kynurenine levels included patients who provided blood samples on C1D1 and C2D1. Paired T tests within each treatment arm were used for comparisons of circulating kynurenine levels. The cutoff date for these analyses was August 15, 2018.

## Results

### Participants

**Fig. 1:**
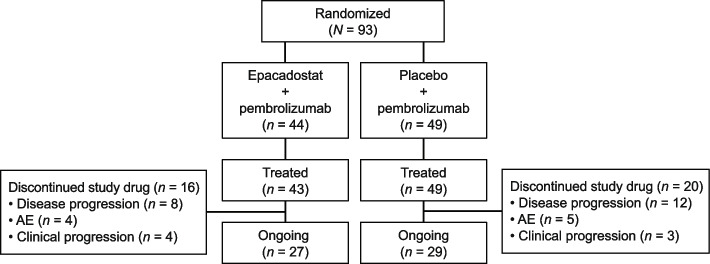
Patient disposition. *AE* adverse event

A total of 93 cisplatin-ineligible patients with advanced UC were randomized (epacadostat plus pembrolizumab, *n* = 44; placebo plus pembrolizumab, *n* = 49) (Fig. 1). One patient randomized to epacadostat plus pembrolizumab was not treated. In both treatment arms, the most common reason for study drug discontinuation was disease progression. Patients with ongoing clinical benefit could continue study treatment (per investigator discretion), and at data cutoff, 62.8% and 59.2% of treated patients were receiving open-label epacadostat plus pembrolizumab or pembrolizumab, respectively.

Most patients presented with metastatic disease (epacadostat plus pembrolizumab, 86.4%; placebo plus pembrolizumab, 91.8%), and approximately half had tumors with PD-L1 CPS ≥ 10 (56.8% and 55.1%, respectively) (Table 1). In the epacadostat-plus-pembrolizumab arm, the median (range) duration of exposure to each agent was 86 (6–189) and 85 (1–171) days, respectively. In the control arm, the median (range) duration of exposure to placebo and pembrolizumab was 85 (3–167) and 85 (1–167) days, respectively. The median duration of follow-up was 64 days in both arms.

**Table 1:** Patient demographics and disease characteristics

	Epacadostat + pembrolizumab (*n* = 44)	Placebo + pembrolizumab (*n* = 49)
Male, *n* (%)	33 (75.0)	38 (77.6)
Median age, years (range)	74.0 (51–90)	72.0 (50–88)
Age ≥ 65 years, *n* (%)	35 (79.5)	40 (81.6)
Race, *n* (%)		
White	33 (75.0)	37 (75.5)
Asian	9 (20.5)	8 (16.3)
Unknown	2 (4.5)	4 (8.2)
ECOG performance status score^a^		
0	8 (18.2)	12 (24.5)
1	15 (34.1)	18 (36.7)
2	21 (47.7)	19 (38.8)
Disease status at screening, *n* (%)		
Locally advanced/unresectable	6 (13.6)	4 (8.2)
Metastatic	38 (86.4)	45 (91.8)
Metastases location, *n* (%)		
Visceral disease	27 (61.4)	35 (71.4)
Lymph node only	12 (27.3)	8 (16.3)
Neither visceral disease nor lymph node only	5 (11.4)	6 (12.2)
Liver metastases present, *n* (%)	5 (11.4)	11 (22.4)
Primary tumor location, *n* (%)		
Upper tract	8 (18.2)	9 (18.4)
Lower tract	36 (81.8)	35 (71.4)
Unknown	0	5 (10.2)
Prior neoadjuvant/adjuvant platinum-based chemotherapy, *n* (%)	6 (13.6)	6 (12.2)
Prior BCG therapy, *n* (%)	2 (4.5)	4 (8.2)
Bajorin risk score		
0	7 (15.9)	9 (18.4)
1	27 (61.4)	25 (51.0)
2	10 (22.7)	15 (30.6)
PD-L1 status, *n* (%)		
CPS ≥ 10	25 (56.8)	27 (55.1)
CPS < 10	19 (43.2)	22 (44.9)
Primary reason for cisplatin-ineligibility,^b^ *n* (%)		
ECOG performance status score ≥ 2	20 (45.5)	15 (30.6)
Creatinine clearance < 60 mL/min	13 (29.5)	20 (40.8)
Grade ≥ 2 audiometric hearing loss	3 (6.8)	5 (10.2)
Grade ≥ 2 peripheral neuropathy	2 (4.5)	2 (4.1)
NYHA class III heart failure	1 (2.3)	2 (4.1)
Multiple reasons	5 (11.4)	4 (8.2)
Missing	0	1 (2.0)

*BCG* Bacillus Calmette-Guérin, *CPS* combined positive score, *ECOG* Eastern Cooperative Oncology Group, *NYHA* New York Heart Association, *PD-L1* programmed death-ligand 1

^a^Assessed during screening

^b^Assessed by the study investigator during screening

### Response rates

**Fig. 2:**
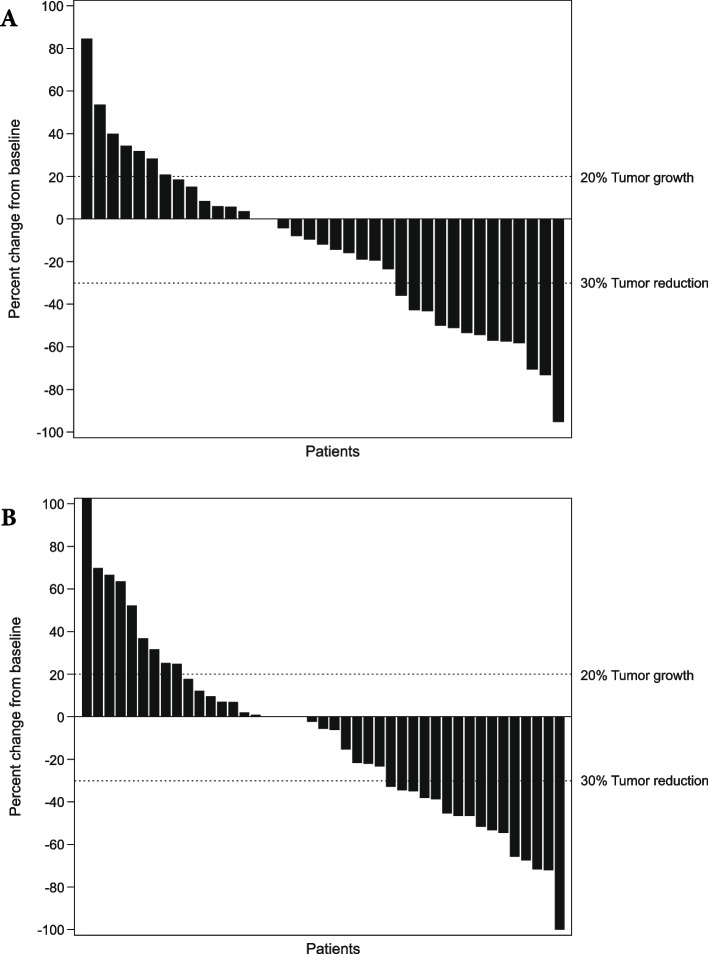
Maximum percentage change from baseline in tumor size per investigator assessment (intent-to-treat analysis). **a** Epacadostat plus pembrolizumab. **b** Placebo plus pembrolizumab

Based on all available data at cutoff, ORR (unconfirmed) was 31.8% (95% CI, 22.46–55.24%) for epacadostat plus pembrolizumab and 24.5% (95% CI, 15.33–43.67%) for placebo plus pembrolizumab (Table 2). The corresponding values based on data from the week 9 visit only were 27.3% and 20.4% (Supplementary Table 1). Waterfall plots for the best change in target lesion size from baseline using all available data at cutoff and data from the week 9 visit only are summarized in Fig. 2 and Supplementary Fig. 1, respectively.

**Table 2:** Investigator-assessed best overall response per RECIST version 1.1 (intent-to-treat analysis)^a^

*n*, (%)	Epacadostat + pembrolizumab (*n* = 44)	Placebo + pembrolizumab (*n* = 49)
ORR^b^ [95% CI^c^]	14 (31.8) [22.46–55.24]	12 (24.5) [15.33–43.67]
Complete response	2 (4.5)	1 (2.0)
Partial response	12 (27.3)	11 (22.4)
Stable disease	13 (29.5)	10 (20.4)
Progressive disease	10 (22.7)	20 (40.8)
No assessment^d^	7 (15.9)	7 (14.3)

*CI* confidence interval, *ORR* objective response rate, *RECIST* Response Evaluation Criteria in Solid Tumors

^a^Based on all available data at cutoff; responses were unconfirmed

^b^Includes patients with an unconfirmed complete or partial response

^c^Per the Clopper-Pearson exact method

^d^Includes patients with a baseline but no post-baseline assessment, including those who discontinued or died before the first post-baseline scan

ORR was also assessed by PD-L1 status. Based on all available data at cutoff, the ORR (unconfirmed) was 26.3% (5/19) for epacadostat plus pembrolizumab and 31.8% (7/22) for placebo plus pembrolizumab among patients with CPS < 10. The corresponding ORRs for patients with CPS ≥ 10 were 36.0% (9/25) and 18.5% (5/27) in the epacadostat-plus-pembrolizumab and placebo-plus-pembrolizumab groups, respectively.

### Safety and tolerability

The rates of AEs, including treatment-emergent grade ≥ 3 AEs and treatment-related grade ≥ 3 AEs, were similar in both treatment arms (Table 3). Immune-related AEs occurred in seven patients in the epacadostat-plus-pembrolizumab group and in five patients in the placebo-plus-pembrolizumab group. Treatment-emergent serious AEs were reported in 13 patients in each treatment arm. The only serious AEs reported in more than one patient in a treatment arm were urinary tract infection (epacadostat plus pembrolizumab, *n* = 3; placebo plus pembrolizumab, *n* = 4) and acute kidney injury (placebo plus pembrolizumab, *n* = 2). Three patients in the epacadostat-plus-pembrolizumab arm experienced a treatment-related serious AE (left ventricular dysfunction, encephalitis, herpes zoster). Five patients in the placebo-plus-pembrolizumab arm experienced a treatment-related serious AE (Huntington's disease, cholestatic hepatitis, infusion-related reaction, autoimmune nephritis, interstitial lung disease).

**Table 3:** Safety summary (as-treated analysis)^a^

Patients, *n* (%)	Epacadostat + pembrolizumab (*n* = 43)	Placebo + pembrolizumab (*n* = 49)
Any AE	39 (90.7)	43 (87.8)
Treatment-related AE	25 (58.1)	29 (59.2)
Grade ≥ 3 AE	22 (51.2)	20 (40.8)
Treatment-related	9 (20.9)	7 (14.3)
Serious AE	13 (30.2)	13 (26.5)
Treatment-related	3 (7.0)	5 (10.2)
Discontinued study drug due to an AE	6 (14.0)	7 (14.3)
Treatment-related	5 (11.6)	2 (4.1)
Discontinued study drug due to a serious AE	3 (7.0)	4 (8.2)
Treatment-related	2 (4.7)	1 (2.0)
Death	2 (4.7)	1 (2.0)
Treatment-related	0	0

The relatedness of an AE to study drug was determined by the investigator. “Discontinued study drug due to an AE” means that ≥ 1 study drug was discontinued due to an AE

*AE*, adverse event

^a^Non-serious AEs up to 30 days of last dose and serious AEs up to 90 days of last dose are included

In total, 11.6% of patients in the epacadostat-plus-pembrolizumab arm compared with 4.1% in the placebo-plus-pembrolizumab arm discontinued study drug due to a treatment-related AE. No treatment-related AE resulted in death.

### Pharmacodynamic activity of epacadostat

**Fig. 3:**
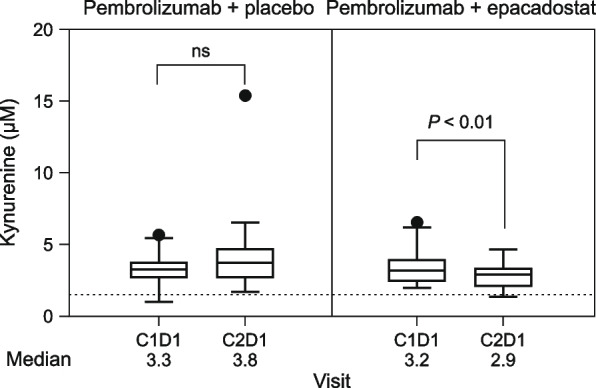
Pharmacodynamic effect of epacadostat 100 mg twice daily dosing as shown by change from baseline in circulating kynurenine levels. The number of samples assessed was 43 in the placebo plus pembrolizumab group (36 for C2) and 34 in the epacadostat plus pembrolizumab group. Statistical analyses were conducted using paired t-tests within each treatment arm. The dotted line indicates the median kynurenine level in healthy subjects (1.5 μM) [25]. *C* cycle, *D* day, *ns* not significant

Circulating kynurenine levels at baseline (C1D1) and after one cycle of treatment (C2D1) are shown in Fig. 3. Compared with baseline, median kynurenine levels at C2D1 were numerically higher in the placebo-plus-pembrolizumab arm (3.3 µM vs. 3.8 µM) and were lower in the epacadostat-plus-pembrolizumab arm (3.2 µM vs. 2.9 µM). Median kynurenine levels remained above the median level observed in healthy subjects (1.5 µM) [25] at each time point and across both treatment arms.

## Discussion

Based on the available clinical data, the clinical benefit of targeting both IDO1 and PD-(L)1 in patients with advanced UC or other solid tumors has not been demonstrated. The response rates in the present study are comparable to the confirmed ORR (29%) observed in the phase II KEYNOTE-052 study, which explored first-line treatment with pembrolizumab monotherapy in cisplatin-ineligible patients with advanced UC [12]. In the phase III ECHO-301/KEYNOTE-252 study in patients with unresectable or metastatic melanoma, no statistically significant differences with the addition of epacadostat to pembrolizumab were found on the dual primary endpoints (median PFS: 4.7 vs. 4.9 months, one-sided *P* = 0.52; median OS, not reached in either arm) after a median duration of follow-up of 12.4 months [28]. Combination treatment with the IDO1 inhibitor navoximod and the PD-L1 inhibitor atezolizumab was assessed in a phase I study of patients with advanced solid tumors, including UC. The regimen was tolerable and antitumor activity was seen, but the benefit of adding navoximod to atezolizumab was not apparent [29]. The IDO1 inhibitor BMS-986205 in combination with nivolumab is being evaluated in a phase I/IIa trial for patients with solid tumors. Among patients with advanced urothelial cancer with no prior immune-oncology therapy (*n* = 27), there was evidence of activity (ORR, 37%) at a median follow up of 24 weeks [30].

Going forward, the pharmacodynamics results from our study suggest that exploration of higher doses of epacadostat are warranted. In contrast to previously reported results for epacadostat monotherapy at doses of 100 mg or higher [25], treatment with epacadostat 100 mg twice daily in combination with pembrolizumab did not lead to complete normalization of circulating kynurenine levels in our study. A numerical, though not statistically significant, increase from baseline in kynurenine levels was also observed in the pembrolizumab/placebo arm. These results suggest that higher doses of epacadostat may possibly be needed to fully suppress kynurenine production. This hypothesis is supported by findings from a retrospective analysis showing that doses of epacadostat ≥ 600 mg BID were needed to durably control kynurenine production when administered with a checkpoint inhibitor [31]. Epacadostat plus pembrolizumab was generally tolerable in this patient population, with a safety profile comparable to that of pembrolizumab monotherapy. No new safety concerns were identified, although the proportion of patients who discontinued study drug due to a treatment-related AE was higher with the combination regimen (11.6% vs. 4.1%). Other avenues of future study include evaluation of additional biomarkers, which may assist in the identification of patients most likely to benefit from combined inhibition of IDO1 and PD-(L)1 [32]. In addition, treatment with IDO1 inhibition may potentially be more effective earlier in the disease course. The phase III ENERGIZE trial (NCT03661320) is currently investigating IDO1 inhibition in combination with nivolumab before and after radical cystectomy in patients with muscle-invasive bladder cancer. The phase II CheckMate 9UT trial (NCT03519256) is investigating nivolumab monotherapy and combinations with IDO1 inhibition, with bacillus Calmette-Guerin (BCG), or the triple combination for patients with BCG-unresponsive, non-muscle invasive bladder cancer [33].

Our understanding of the role of immune checkpoint inhibition in the first-line treatment of cisplatin-ineligible patients with advanced/metastatic UC is rapidly evolving. At the time the current study was designed, the rationale for the pembrolizumab monotherapy control arm in cisplatin-ineligible patients was based on promising results from the single-arm phase II KEYNOTE-052 study, [11] which supported the accelerated approval of this agent in the US. The recent DANUBE study investigated durvalumab alone, durvalumab plus tremelimumab, or chemotherapy in previously untreated unresectable, locally advanced or metastatic urothelial cancers and included patients who were cisplatin-ineligible [34]. The authors noted relatively similar OS among cisplatin-ineligible and eligible patients in each treatment group, while they also suggested that CTLA-4 inhibition did not add significant clinical benefit to PD-L1 inhibition in the first-line setting [34].

The addition of immune checkpoint inhibition to platinum-based chemotherapy has also been tested in two recent studies in patients with advanced/metastatic UC. In the randomized phase III KEYNOTE-361 study, the addition of pembrolizumab to cisplatin- or carboplatin-based chemotherapy yielded numerically longer PFS and OS, but this did not reach the prespecified thresholds for statistical significance [35]. In the randomized phase III IMvigor130 trial, the addition of atezolizumab to cisplatin- or carboplatin-based chemotherapy significantly improved PFS, but the difference in median PFS was only about 2 months, while there was no significant OS difference [36]. As noted previously, results from the JAVELIN Bladder 100 trial have led to the adoption of a sequential treatment strategy consisting of platinum-based chemotherapy followed by switch maintenance avelumab for patients with no progression on chemotherapy as a new standard of care.

## Conclusions

In this study, combining epacadostat 100 mg twice daily with pembrolizumab resulted in an ORR similar to that of pembrolizumab monotherapy in cisplatin-ineligible patients with previously untreated locally advanced/unresectable or metastatic UC. No new safety concerns were identified, and the safety profile of the combination regimen was similar to that of pembrolizumab plus placebo. Epacadostat 100 mg twice daily did not fully normalize circulating kynurenine levels when administered with pembrolizumab. Firm conclusions based on these results cannot be made because the study was halted early, resulting in a relatively small sample size (*N* = 93) and a short duration of follow-up.

### Supplementary Information

**Additional file 1: Supplementary Table 1.** Investigator-assessed best overall response per RECIST version 1.1 based on data acquired only at the Week 9 visit (intent-to-treat analysis).

**Additional file 2: Supplementary Fig. 1.** Maximum percentage change from baseline in tumor size per investigator assessment per RECIST version 1.1 based on data acquired only at the week 9 visit (intent-to-treat analysis). **a** Epacadostat plus pembrolizumab. **b** Placebo plus pembrolizumab.

## Data Availability

Access to individual patient-level data is not available for this study.

## Abbreviations

AE: Adverse event
BCG: Bacillus Calmette-Guérin
C: Cycle
CI: Confidence interval
CPS: Combined positive score
D: Day
ECOG: Eastern Cooperative Oncology Group
IDO1: Indoleamine 2,3- dioxygenase 1
NS: Not significant
NYHA: New York Heart Association
ORR: Objective response rate
OS: Overall survival
PD-L1: Programmed death-ligand 1
PFS: Progression-free survival
RECIST: Response Evaluation Criteria in Solid Tumors
UC: Urothelial carcinoma
