# Supplementary material for: Pembrolizumab plus either epacadostat or placebo for cisplatin-ineligible urothelial carcinoma: results from the ECHO-307/KEYNOTE-672 study
Source: BMC Cancer. 2024 Jul 25;23(Suppl 1):1252. doi: 10.1186/s12885-023-10727-3 (PMC11270764; doi:10.1186/s12885-023-10727-3)
Supplement: Supplementary file 1 — Additional file 1: Supplementary Table 1. Investigator-assessed best overall response per RECIST version 1.1 based on data acquired only at the Week 9 visit (intent-to-treat analysis). [file 12885_2023_10727_MOESM1_ESM.docx]

**Supplementary Table 1** Investigator-assessed best overall response per RECIST version 1.1 based on data acquired only at the Week 9 visit (intent-to-treat analysis)

| *n*, (%) | Epacadostat + pembrolizumab  (*n* = 44) | Placebo + pembrolizumab  (*n* = 49) |
| --- | --- | --- |
| ORR^a^ [95% CI^b^] | 12 (27.3)  [18.01–49.79] | 10 (20.4)  [11.76–38.63] |
| Complete response | 1 (2.3) | 0 |
| Partial response | 11 (25.0) | 10 (20.4) |
| Stable disease | 15 (34.1) | 10 (20.4) |
| Progressive disease | 10 (22.7) | 20 (40.8) |
| No assessment^c^ | 7 (15.9) | 9 (18.4) |

*CI* confidence interval, *ORR* objective response rate, *RECIST* Response Evaluation Criteria in Solid Tumors

^a^Includes patients with an unconfirmed complete or partial response

^b^Per the Clopper-Pearson exact method

^d^Includes patients with a baseline but no post-baseline assessment, including those who discontinued or died before the first post-baseline scan
