# Supplementary figures and images for: Pembrolizumab plus either epacadostat or placebo for cisplatin-ineligible urothelial carcinoma: results from the ECHO-307/KEYNOTE-672 study
Source: BMC Cancer. 2024 Jul 25;23(Suppl 1):1252. doi: 10.1186/s12885-023-10727-3 (PMC11270764; doi:10.1186/s12885-023-10727-3)

**A**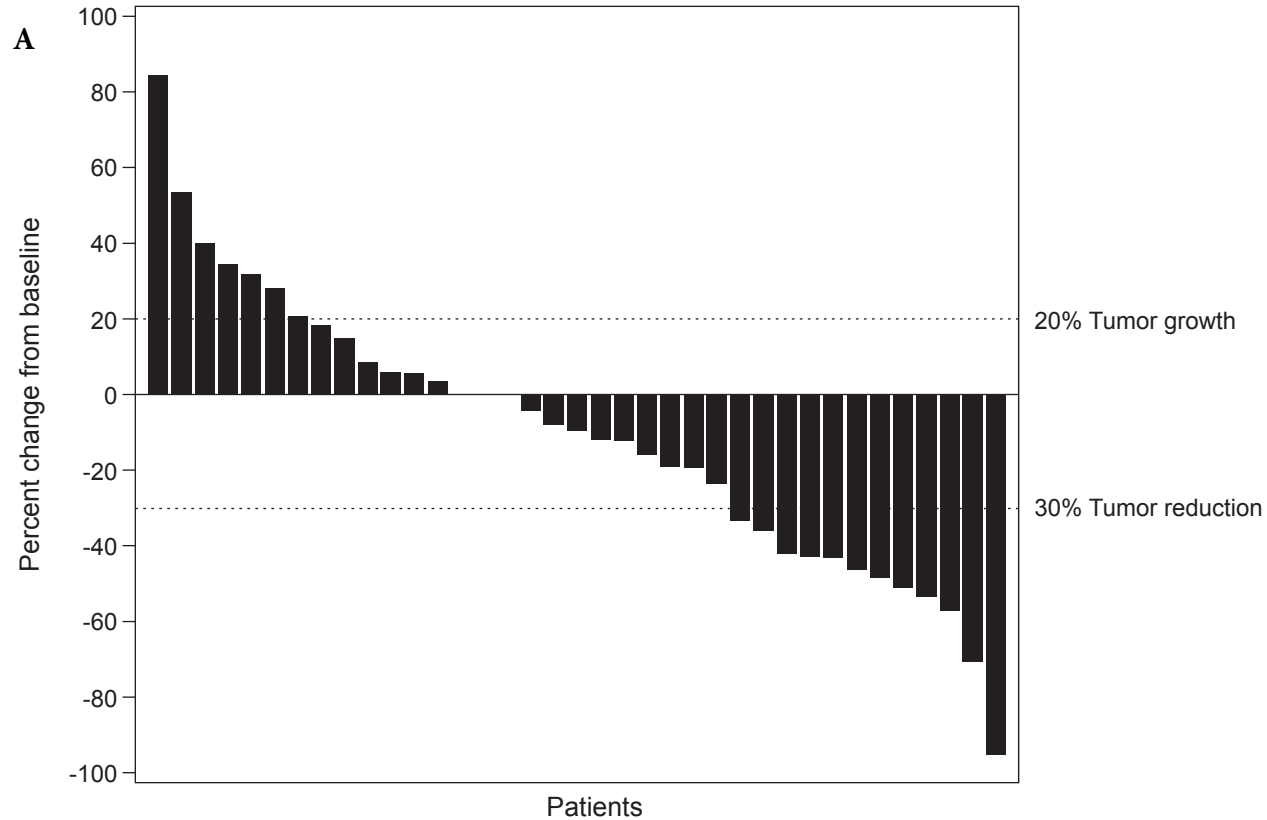

**B**

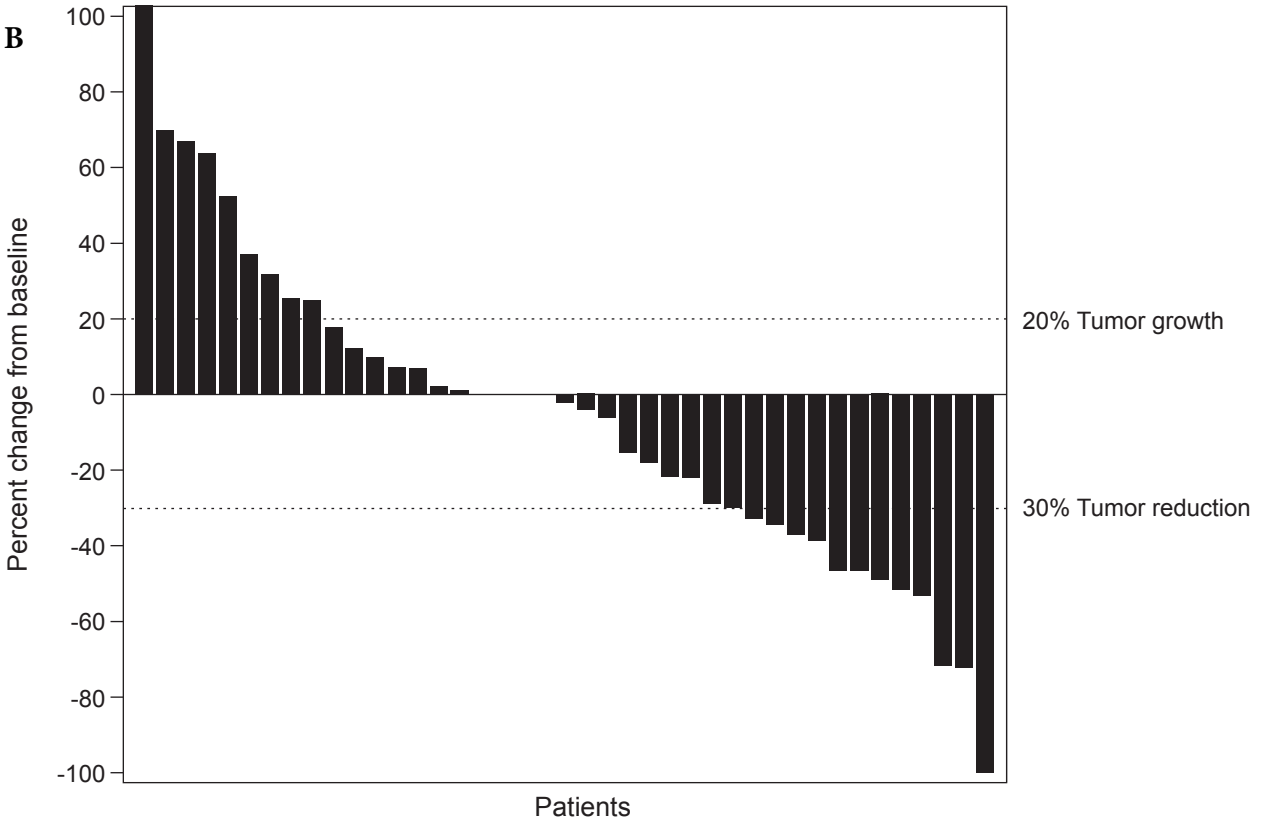

Supplement: Supplementary file 2 — Additional file 2: Supplementary Fig. 1. Maximum percentage change from baseline in tumor size per investigator assessment per RECIST version 1.1 based on data acquired only at the week 9 visit (intent-to-treat analysis). a Epacadostat plus pembrolizumab. b Placebo plus pembrolizumab. [file 12885_2023_10727_MOESM2_ESM.pdf]
